# Challenges of engineering a functional growth plate *in vitro*

**DOI:** 10.3389/fbioe.2025.1550713

**Published:** 2025-03-04

**Authors:** Gangyu Zhang, Adrien Moya, Arnaud Scherberich, Ivan Martin

**Affiliations:** ^1^ Department of Biomedicine, University Hospital Basel, University of Basel, Basel, Switzerland; ^2^ Department of Biomedical Engineering, University of Basel, Allschwil, Switzerland

**Keywords:** growth plate, skeletal stem cell, endochondral ossification, organoids, stem cell niche, bioengineering, chondrocyte

## Abstract

Several cartilage and bone organoids have been developed *in vitro* and *in vivo* using adult mesenchymal stromal/stem cells (MSCs) or pluripotent stem cells (PSCs) to mimic different phases of endochondral ossification (ECO), as one of the main processes driving skeletal development and growth. While cellular and molecular features of growth plate-like structures have been observed through the generation and *in vivo* implantation of hypertrophic cartilage tissues, no functional analogue or model of the growth plate has yet been engineered. Herein, after a brief introduction about the growth plate architecture and function, we summarize the recent progress in dissecting the biology of the growth plate and indicate the knowledge gaps to better understand the mechanisms of its development and maintenance. We then discuss how this knowledge could be integrated with state-of-art bioengineering approaches to generate a functional *in vitro* growth plate model.

## 1 Introduction

The growth plate (GP) is a highly organized hyaline cartilage tissue located at the metaphysis of growing bones, playing a critical role in bone development and growth through the process of endochondral ossification (ECO) (Mackie et al., 2011). During embryogenesis, ECO begins with the condensation of embryonic mesenchymal stromal/stem cells (MSCs), which subsequently undergo chondrogenic differentiation to form a cartilage template. As the cartilage mold expands, the chondrocytes at the center cease proliferating and become hypertrophic, releasing catabolic cytokines and angiogenic factors for the recruitment of endothelial cells, osteoblasts and osteoclasts. In turn, these cells invade the cartilaginous structure, lay down bone-like matrix and form the primary ossification center (POC) (Mackie et al., 2008). Cell proliferation, combined to an increase of cell volume due to hypertrophic enlargement and the neo synthesis and secretion of extracellular matrix (ECM), leads to the elongation of long bones, contributing to the increase in body length during the gestation (Cooper, 2019). Shortly after birth, the secondary ossification center (SOC) forms at the epiphysis, dividing the pre-existing cartilage to form the GP which continues to drive postnatal bone growth via ECO. Injuries or disorders affecting the GP, such as epiphyseal bone fractures, achondroplasia, and cancer, will cause limb length discrepancies (Cass and Peterson, 1983; Rousseau et al., 1994; Shiang et al., 1994; Jesus-Garcia et al., 2000). Since the regeneration ability of the GP is limited due to its avascular nature and complex architecture, immediate attention is necessary to prevent growth disorders following injuries. However, to date, the treatment of growth defects is hindered by the limited knowledge regarding the molecular and cellular mechanisms governing human bone growth, which also restricts the application of tissue engineering (TE) and regenerative strategies. Developing an *in vitro* GP model derived from human cells would provide a valuable platform to study the physiology and pathology of the GP and significantly advance both drug discovery efforts and the development of tissue engineering solutions for growth disorders. Moreover, developing a new paradigm of engineering a cartilage tissue capable of growing in concert with the body and adapting to its changing needs, could inspire innovative tissue engineering strategies rooted in developmental biology and their application in paediatric settings. While several studies have successfully generated cartilage organoids *in vitro* and bone organoids *in vivo* by recapitulating the path of ECO, engineering a functional GP *in vitro* remains an open challenge. In this review, we will summarize some recent findings in the molecular and cellular understanding of the GP and discuss how the development of new fundamental biological knowledge, combined with the adoption of advanced bioengineering tools, could inspire new strategies to engineer a functional GP.

## 2 Organization of the growth plate

**FIGURE 1:**
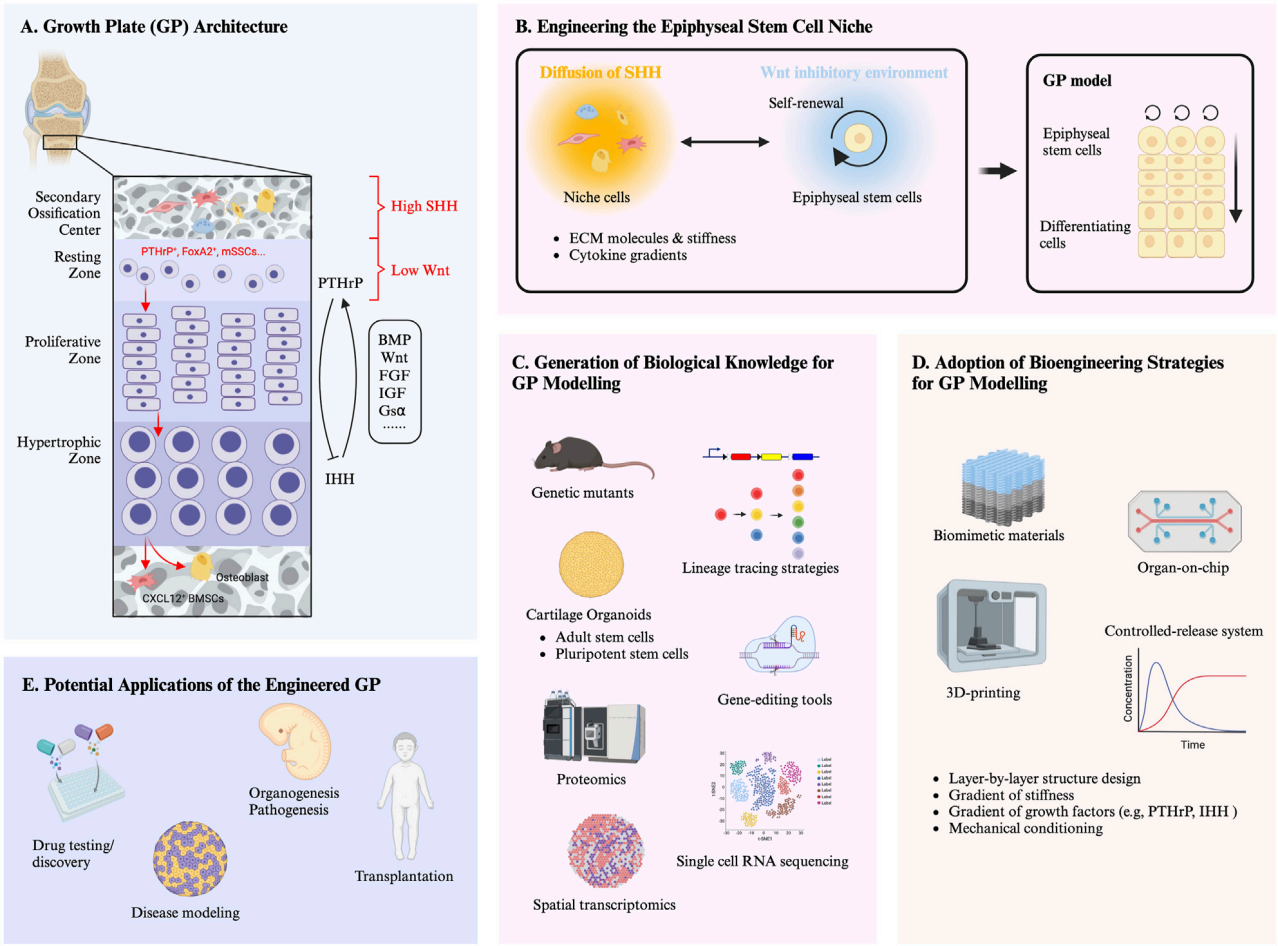
**(A)** Growth plate (GP) architecture. The graphics highlights the key molecular pathways and feedback loops involved in the different zones of the GP, as well as the regulatory molecules involved in the establishment and maintenance of an epiphyseal stem cell niche. The different cell morphologies represent cell populations (i.e., mesenchymal stromal cells, endothelial cells and haematopoietic cells) within the secondary ossification center (SOC). Red arrows indicate the lineage of the skeletal stem cells (SSCs) within the resting zone of GP. **(B)** Engineering the epiphyseal stem cell niche. The graphics highlights that the recapitulation of the functional features of the GP requires the formation of a specific niche. Self-renewing stem cells and accessory cell populations (i.e., mesenchymal stromal cells, endothelial cells and haematopoietic cells) are in charge of an orchestrated integration of biochemical and biophysical signals (e.g., extracellular matrix (ECM) molecules and stiffness, cytokine gradients). **(C)** Generation of biological knowledge for GP modelling. Deeper understanding of molecular and cellular mechanisms underlying GP development by utilizing traditional and innovative tools (e.g., genetic mutants, lineage tracing, gene-editing, organoid systems, and transcriptomic/proteomic) will be key to recapitulate the epiphyseal stem cell niche and thus to engineer models of a functional GP. **(D)** Adoption of bioengineering strategies for GP modelling. Bioengineering tools, such as biomimetic materials, controlled-release systems, 3D printing, and Organ-on-chip, hold the potential to control stem cell behavior and replicate the epiphyseal stem cell niche, towards modelling a functional GP. **(E)** Potential applications of engineered growth plate. The development of a functional *in vitro* GP has several potential applications. For example, it could provide a platform for drug testing and discovery as well as studying organogenesis and pathogenesis of GP development. Additionally, it could serve as a graft which could be implanted to treat growth defects. Furthermore, it will provide a paradigm for generating growing tissues and open multiple avenues in paediatric regenerative medicine.

Three distinct layers of chondrocytes can be identified in the GP based on their location, morphology and function: resting chondrocytes, proliferating chondrocytes, and hypertrophic chondrocytes (Figure 1A) (Hallett et al., 2019). Resting chondrocytes have long been considered to possess stem cell properties. In the early 2000s, a ground breaking study using a surgical transplantation model revealed that stem-like cells in the resting zone of the rabbit growth plate can generate clones of proliferating chondrocytes that gradually regenerate the entire growth plate (Abad et al., 2002). These findings highlight the essential role of resting chondrocytes in the formation and structural organization of the GP. In the proliferating zone, chondrocytes become highly proliferative and align into columns parallel to the axis of bone elongation to facilitate the demands of bone growth (Mackie et al., 2011). Subsequently, the chondrocytes exit the cell cycle and undergo hypertrophic differentiation, expressing Collagen Type X and Indian Hedgehog (IHH). The hypertrophic zone interacts with the resting zone via a Parathyroid hormone-related protein (PTHrP)-IHH negative feedback loop, which orchestrates the organization, proliferation and differentiation of GP chondrocytes (Kronenberg, 2003). Briefly, the resting chondrocytes secrete PTHrP which acts on proliferative chondrocytes to keep them proliferating and delays their hypertrophic differentiation. When chondrocytes are distant from the resting zone, they stop proliferating and produce IHH which in turn stimulates the production of PTHrP in the resting zone of the epiphyseal GP (Kronenberg, 2003). The interactions of chondrocytes from different zones of the GP play a critical role in the maintenance of the GP and the regulation of long bone growth. Several cell-signaling pathways like the fibroblast growth factor (FGF) family (Naski et al., 1998; Zhou et al., 2015), insulin-like growth factor (IGF) (Wang et al., 2011), G-protein alpha subunit (Gsα)-mediated signaling (Sakamoto et al., 2005; Regard et al., 2013; Bastepe et al., 2004), Wnt signaling (Guo et al., 2009; Später et al., 2006; Hu et al., 2005) and Transforming growth factor β (TGF-β)/Bone morphogenetic protein (BMP) signaling (Hojo et al., 2013; Minina et al., 2001; Kameda et al., 1999; Wu et al., 2016) have been demonstrated to potentially interact with the PTHrP-IHH negative feedback loop to orchestrate the proper function of diverse cell types within the GP [for review see ref (Samsa et al., 2017)].

## 3 Growth plate skeletal stem cells and postnatal long bone growth

The concept of “mesenchymal stromal/stem cells (MSCs)” originates from the pioneering experiments demonstrating that heterotopic bone marrow transplantation could generate ectopic bone and bone marrow (Friedenstein et al., 1966; Tavassoli and Crosby, 1968). The term “MSCs” was later used to indicate cell populations with the capacity for plastic-adherent and tri-lineage differentiation, as well as the expression of a group of immunophenotypic surface markers (Castro-Malaspina et al., 1980; Owen and Friedenstein, 1988; Caplan, 1991; Dominici et al., 2006). However, MSCs fulfilling such minimal criteria are highly heterogeneous and often fail to display stem cell properties, as well as to form chondro-osseous organs supporting haematopoiesis *in vivo*. Therefore, the term “skeletal stem cells (SSCs)” was introduced to define more specific populations of self-renewing and multipotent cells that reside in the skeletal system (Bianco et al., 2013; Bianco and Robey, 2015). Over the decades, the identification of SSCs has increasingly gained attention from bone researchers worldwide [for review see refs (Li et al., 2022; Serowoky et al., 2020)].

Using the *Cre-loxP* system driven lineage tracing strategy, several SSCs have been identified in the mouse GP. The study of Noriaki et al. demonstrated that Sox9^+^, Col2^+^, and Acan^+^ chondroprogenitors are the major source of chondrocytes, osteoblasts, stromal cells, and bone marrow stromal/mesenchymal progenitor cells during endochondral bone development (Ono et al., 2014). In 2018, Mizuhashi et al. showed that a subset of PTHrP^+^ chondrocytes in the resting zone phenotypically and functionally behave as SSCs, as evidenced by their expression of surface markers partially overlapping with the CD51^+^CD90^−^CD105^−^CD200^+^ mouse SSCs and their capacity to differentiate into proliferative-, hypertrophic-chondrocytes, Cxcl12-expressing reticular stromal cells and Col1a1-expressing osteoblasts in long term tracing (Mizuhashi et al., 2018). FoxA2^+^ cells are distinct SSCs situated at the interface between the SOC and the resting zone (Muruganandan et al., 2022). Lineage tracing of these cells confirmed their osteogenic and chondrogenic potential during early postnatal stage (P0 - P28) and their chondrogenic potential after P28, contributing to the maintenance of GP turnover and regeneration.

By using fluorescence-activated cell sorting (FACS) and transplantation assays, Chan et al. successfully identified mouse SSCs (CD45-Ter119- Tie2-AlphaV + Thy-6C3-CD105-CD200+) in the GP and plotted their lineage differentiation trajectory (Chan et al., 2015). These cells are capable of self-renewal and give rise to bone, cartilage, and stromal derivatives when transplanted beneath the renal capsule. Then, by comparing the human bone-derived cells to several murine skeletal cell populations, Chan et al. identified human SSCs based on a panel of surface markers (CD45^−^CD235a^−^Tie2^−^CD31^−^PDPN^+^CD146^−^CD73^+^CD164^+^) (Chan et al., 2018). These cells can be isolated from human foetal GP, BMP2-treated adipose stromal cells and induced pluripotent stem cells (iPSCs) and exhibit self-renewal and multipotent potential both *in vitro* and *in vivo*. Single-cell RNA sequencing (scRNA-seq) showed that these PDPN^+^CD146^−^CD73^+^CD164^+^ human SSCs have similar gene expression profiles and promoter accessibilities than the ones previously identified for mouse SSCs (Chan et al., 2015).

Collectively, these findings have developed our understanding of the organization of the GP and of the resident stem cell populations. However, the identification of SSC in different studies is often based on different techniques (e.g., lineage tracing vs. sorting) and the characterization of their functionality is not always assessed using the same standards (e.g., including analysis at clonal level). Thus, a critical and detailed review of the literature is necessary to discriminate possibly different cell populations which are all associated with the same terminology of SSC.

In the mouse GP, a specific stem cell niche has been identified by Newton et al., in 2019 (Newton et al., 2019). Clonal lineage tracing experiments showed that the resting zone chondroprogenitors are able to form longer columns (more than nine cells) during the postnatal life instead of short columns (only a few cells) observed during the foetal and neonatal period. The driving force of this post-natal “switch” is that chondroprogenitors in the resting zone acquire self-renewal ability and undergo symmetric cell division within the so-called SOC. This process, leading to continuous longitudinal bone growth, is driven by high levels of sonic hedgehog (SHH) and is coordinated by the neighbouring mesenchymal stromal cells, endothelial cells and haematopoietic cells.

While these findings have significantly deepened our understanding of the GP, several molecular and cellular mechanisms underlying human GP maintenance and long bone growth remain largely unknown. For example, as of yet, there are no reliable markers for human resting chondrocytes. In addition, despite evidence of stem cell properties for these cells, it is not known whether an epiphyseal stem cell niche has similar features in human and how it regulates cell behavior in the GP resting zone. *In vitro* models of human GP are thus necessary to shed light on the processes orchestrating the GP structure and function.

## 4 Current engineered cartilage organoids to study skeletal development

Tissue engineering holds great promise for developing complex 3D models to address specific questions related to GP biology. Several groups have generated cartilage and bone organoids from both adult stem/stromal cells (ASCs) and pluripotent stem cells (PSCs) by recapitulating developmental mechanisms. Our group and others have shown that human adult MSCs derived from bone marrow and adipose tissue are able to generate hypertrophic cartilage *in vitro* and form an ectopic bone-bone marrow organ *in vivo*, mirroring the developmental processes of ECO (Scotti et al., 2010; Osinga et al., 2016; Farrell et al., 2011; van der Stok et al., 2014; Pelttari et al., 2006; Mumme et al., 2012). The work of Craft et al. and Richard et al. showed that by modulating the differentiation protocol, human PSCs can be differentiated into two distinct human chondrogenic lineages including GP chondrocytes and articular chondrocytes with transcriptomic profiles similar to fetal epiphyseal and GP (Craft et al., 2015; Richard et al., 2023). This study provides valuable insights into the molecular mechanisms underlying human cartilage development and biology. Shireen et al. proposed a protocol that allows direct differentiation of iPSCs-derived sclerotome to hypertrophic chondrocytes with the ability to trans-differentiate into osteoblasts, mirroring endochondral bone development (Lamande et al., 2023). One example of generating a GP with a zonal organization has been offered by Alsberg et al. in an *in vivo* setting (Alsberg et al., 2002). The authors showed that chondrocytes and osteoblasts delivered with RGD-alginate hydrogels into immunodeficient (CB17) mice are able to generate tissues that morphologically and functionally resemble the GP, as evidenced by GP-like structures and their ability to grow in size. Other groups using iPSCs also observed a GP-like structure when they implanted iPSCs-derived hypertrophic cartilage *in vivo* (Loh et al., 2016; Matsuda et al., 2020). All these findings indicate the feasibility to generate a GP-like structure *in vitro* by identifying suitable cells and the key signals that they would receive *in vivo*. In this context, it is important to remark that different cell sources may require specific differentiation protocols and have preferential relevance towards defined applications. For more detailed analysis of these topics, we refer to recent reviews concerning MSCs (Narcisi et al., 2021) and iPSCs (De Kinderen et al., 2022).

All these *in vitro* models only partially recapitulate the GP phenotypes and functions. In fact, while the engineered cartilages share similar gene expression profiles with the native tissues at the bulk RNA level, their transcriptomic landscape at single-cell resolution, particularly across temporal and spatial dimensions, remains largely unexplored. There is also no evidence of the presence of stem-like cells within these engineered constructs and of the maintenance of a stem cell pool over time. In addition, the zonal organization, which is key to a functional GP, is absent in all of these *in vitro* organoids, and it is unclear if it would be acquired and maintained to sustain bone growth after implantation *in vivo*.

A functional engineered GP should ideally: (i) harbor resting, proliferating, and hypertrophic chondrocytes which communicate with each other to support tissue homeostasis, (ii) maintain proper signaling activity such as IHH/PTHrP, BMP, WNT, and FGFs and responsiveness to molecular and mechanical environmental cues, and (iii) enable chondrocyte proliferation and matrix production, ultimately contributing to controlled bone elongation during the process of ECO. Such a functional GP model would serve as a physiologically relevant platform for drug testing and discovery, towards development of therapeutics targeting skeletal growth disorders. It would also facilitate personalized medicine approaches by allowing patient-specific studies of skeletal diseases and treatment responses. Moreover, it would offer a powerful tool for studying human skeletal development, to investigate molecular mechanisms underlying chondrogenesis, ossification, and longitudinal bone growth in controlled and human settings. Following is a more detailed analysis of the developments required to reach this goal, along with the envisioned associated challenges to address.

## 5 Expected developments and potential challenges for engineering a functional growth plate

### 5.1 Identifying the pivotal components of a growth plate stem cell niche, towards its *in vitro* modelling

The concept of a “niche” was first introduced in the context of haematopoiesis by Raymond Schofield in 1978 to describe how hematopoietic stem cells (HSCs) maintain their stemness within a specific microenvironment (Schofield, 1978). Several stem cell niches have been identified in mammalian systems including the hematopoietic stem cell niche (Crane et al., 2017), epithelial stem cell niche (Tumbar et al., 2004), intestinal stem cell niche (McCarthy et al., 2020), and neural stem cell niche (Miller and Gauthier-Fisher, 2009). These niches have been shown to regulate stem cell behavior by providing extrinsic biochemical and biophysical signals. Engineering specialized environments will provide enhanced control over the growth and differentiation of stem cells *in vitro*. For example, by providing signals regulating intestinal stem cells (ISCs) behavior, single Lgr5^+^ ISCs are able to generate intestinal organoids with all the differentiated cell types and crypt-villus structure (Sato et al., 2009). Inspired by this work, we postulate that engineering the epiphyseal stem cell niche will be essential for developing a functional GP (Figure 1B). However, knowledge about the stem cell niche in the GP is rather limited to date and the main findings in this context are restricted to SHH and WNT signaling, as described below.

The SOC, neighbouring the resting zone of the GP, has been shown to play a critical role in the development of a stem cell niche in the postnatal epiphyseal GP (Newton et al., 2019). The authors showed that the chondroprogenitors within the resting zone acquire self-renewal ability in conjunction with the formation of the SOC, where SHH is released, leading to the maximal extent of hedgehog (HH) signaling specifically in the resting zone. Genetic activation of HH pathway in the PTHrP^+^ resting chondrocytes increase their proliferation rate and leads to the clonal expansion of cells within the resting zone (Orikasa et al., 2024). Implantation of beads containing Smoothened agonist (SAG), a pharmacological activator of HH signaling, into the SOC of Wistar-Kyoto rats, effectively increases longitudinal bone growth (Trompet et al., 2024). All these studies suggest that HH signalling is critical in the maintenance of the stem cell niche in the resting zone. Therefore, presentation of SHH should be considered as pivotal component in the *in vitro* model system.

Wnt signaling also plays a key role in the maintenance and differentiation of resting chondrocytes. Shawn et al. successfully isolated slow-cycling chondrocytes from the resting zone by using a chondrocyte-specific label-retention approach and identified significant upregulation of inhibitors for Wnt signaling in the resting zone by performing comparative RNA-seq analysis (Hallett et al., 2021). The number of PTHrP^+^ resting chondrocytes was decreased and their ability to form columnar chondrocytes was impaired when Wnt/beta-catenin signaling was activated, suggesting that the PTHrP^+^ skeletal stem cells are maintained in a Wnt-inhibitory environment within the resting zone. The study of Dana et al., also showed that the expansion of a stem cell pool following SAG administration is the consequence of the establishment of a Wnt-inhibitory environment (Trompet et al., 2024). Thus, developing a low Wnt environment using specific molecules would be beneficial for engineering a functional resting zone and for the maintenance of stem-like cells *in vitro*.

These findings represent a solid basis to characterize the GP niche. However, further studies are necessary to identify specific cellular phenotypes, molecular pathways, ECM molecules and biophysical factors underlying the development and maintenance of this niche.

### 5.2 Dissecting the molecular and cellular mechanisms underlying growth plate development

The adoption of murine genetic mutants, lineage tracing strategies, gene-editing tools, organoid systems, and transcriptomic/proteomic technologies have greatly broadened and deepened our understanding of skeletal development, homeostasis, and regeneration. Similar approaches and strategies should be introduced also to generate new knowledge on the molecular and cellular mechanism underlying GP development (Figure 1C). This will be key in any endeavour to recapitulate the epiphyseal stem cell niche and thus to engineer models of a functional GP. A few studies have already taken the first steps in this direction, predominantly using single-cell RNA sequencing and spatial transcriptomic, as described below.

Development and application of single cell transcriptome sequencing have led to better understanding of the mouse limb developmental biology. Li et al. have developed an unsupervised bioinformatics approach called “Sinova” that successfully reconstructed a temporal and spatial map of mouse developing GP based on the single-cell RNA-seq data (Li et al., 2016). This model allows systematic discovery of marker genes, signalling pathways, and transcriptional factors regulating GP development, as well as the identification of surface markers to sort cell subpopulations from GP. By analysing 19,952 single cells from murine postnatal limbs, Gao et al. decoded the postnatal GP atlas and identified three different evolutional branches marked by Col10a1, Spp1, and Tnni2 in the growth plate (Gao et al., 2023). Interestingly, the distribution pattern of Tnni2 is similar to that of Cd31^h^Emcn^h^ vessels, indicating that Tnni2^+^ chondrocytes may play a key role in regulating the developmental activity of ossification centres, especially the secondary ossification. These findings, in conjunction with the study of Newton et al—which highlights the critical role of SOC in the maintenance of a stem cell pool within the resting zone—raise the hypothesis that Tnni2^+^ chondrocytes may play crucial roles in the development of the epiphyseal stem cell niche. However, more functional and lineage tracing experiments should be conducted in the future to clarify the characteristics of these cell subpopulations.

Single cell RNA sequencing (scRNA-Seq) has also been applied to dissect embryonic skeletal stem cell ontogeny during early human skeletogenesis. In 2021, He et al. generated the first cell transcriptome atlas of human limb buds and embryonic long bones at single-cell resolution (He et al., 2021). In this study, a novel embryonic skeletal stem/progenitor cell (eSSPC) marked by PDGFRA^low/–^PDPN^+^CADM1^+^ was identified in the perichondrial regions of embryonic long bones. Similar to the previously identified human GP SSCs, the eSSPCs display self-renewal capacity and osteo-chondrogenic potentials both *in vitro* and *in vivo*. Spatial transcriptomic is a more advanced technique that not only allows the measurement of the transcriptome of cells of interest but also of their neighbouring cells, which influence cell phenotypes and functions via cell-cell communication. The first detailed spatiotemporal transcriptomic landscape of human embryonic limb has been generated by Zhang et al., in 2023 using single-cell and spatial transcriptomics (Zhang et al., 2023). This study has enhanced our understanding of tissue architecture, cellular heterogeneity, cell-fate decisions, and cell-cell communication within the developing limb across time and space.

Such technologies hold great potential for advancing our understanding of postnatal limb development. Decades of works have shown that chondrocytes from different zones exhibit distinct transcriptomic profiles. Analysis of the transcriptional landscape of chondrocytes from each zone at single cell resolution during the postnatal stage would significantly enhance our understanding about the cellular heterogeneity of the GP and the mechanisms underlying postnatal long bone growth. In addition, neighbouring cells have been shown to play a critical role in the regulation of resting zone chondrocytes. For example, periosteal stem cells control GP stem cells activity by locally secreted IHH (Tsukasaki et al., 2022). The formation of SOC leads to the development of a stem cell niche and regulates resting chondrocytes behaviours (Newton et al., 2019). Therefore, extending the knowledge gained from single cells to multicellular neighbourhoods will be key to ultimately understand the molecular mechanisms of complex biological systems in the GP.

### 5.3 Adopting bioengineering strategies for modelling the epiphyseal stem cell niche

In parallel with the generation of fundamental knowledge on the biological mechanisms underlying the development of the GP and its stem cell niche, it will be necessary to identify the required engineering tools for its implementation into viable models. Advances in bioengineering, such as biomimetic materials, controlled-release systems, 3D printing and Organ-on-a-chip technologies, have enabled better control over the behavior and organization of organoids. By integrating these novel approaches, it will be possible to engineer an *in vitro* GP that resembles *in vivo* architecture and function (Figure 1D).

ECM is one of the main components of the stem cell niche which not only provides structural support but also controls stem cell function by delivering biochemical and signals and transducing mechanical forces. Accumulating evidences have shown that biophysical cues have crucial impacts on guiding stem cell fate (Huebsch et al., 2015; Das et al., 2016; Chaudhuri et al., 2016). For example, MSCs cultured in a hydrogel with a low stress stiffening induced preferentially adipocytic commitment while osteogenesis was progressively favoured over adipogenesis with increasing stress stiffening (Das et al., 2016). In addition, MSCs can respond to diverse dynamic mechanical forces [e.g., tensile stress (Gao et al., 2011; Ghazanfari et al., 2009; Gong and Niklason, 2008) or compressive stress (Huang et al., 2004; Panadero et al., 2016)] and can be directed into specific differentiation pathways. Several studies have shown that the ECM components and mechanical properties vary across the different zones of the GP and play crucial roles in regulating chondrogenic behaviour. For example, collagen type II is predominantly found in the lower proliferative zone and upper hypertrophic zone of the GP, while collagen type X is only present around hypertrophic chondrocytes (O'Keefe et al., 1994; Sandberg and Vuorio, 1987). Differences in the mechanical properties among different zones have also been evaluated by several groups in animal models (Eckstein et al., 2022; Fischenich et al., 2022). Therefore, it would be valuable to develop or adopt suitable biomaterials mimicking this distribution of ECM components and the gradients of stiffness to direct cells behavior and tissue organization in the different zones of the GP.

Growth factors are important for the development and maintenance of GP *in vivo*. Traditional *in vitro* culture systems rely on the continuous supplementation of growth factors in the culture medium. However, these systems have several limitations, including a lack of control over the spatial distribution of factors, inadequate gradient concentrations, and limited ability to mimic the complex *in vivo* environment. Controlled-release systems encapsulating bioactive molecules within carriers like hydrogels or microspheres enable the delivery of growth factors and cytokines with precise temporal and spatial control (Yang et al., 2022; Vo et al., 2012; Ye et al., 2022). For example, as mentioned above, the gradients of morphogens such as PTHrP and IHH are essential for the organization of the GP and for directing the fate of chondrocytes within the different zones. Therefore, developing materials with concentration gradients of PTHrP and IHH could regulate chondrocyte biology and promote a GP-like structure.

Layer-by-layer 3D printing and cell-laden fibre techniques have been used to generate complex structures such as liver (Snyder et al., 2011), heart (Zhang et al., 2016), vascular networks (Wu et al., 2011; Miller et al., 2012), and osteochondral unit (Santos-Beato et al., 2022). These methods may enhance manufacturing efficiency by enabling the simultaneous printing of multiple tissue layers and allow for volumetrically defined incorporation of different cell types or growth factors, creating tissues or organs that more closely resemble the composition and function of natural counterparts (Freeman et al., 2020). Given that the GP consists of different zones, such technique would be greatly beneficial in the development of *in vitro* models with precise cellular organization and compartmentalization, along with immobilized signalling epitopes.

Microfluidic devices like organ-on-a-chip models can closely mimic the essential physiological and structural features of small functional units of organs, offering a platform for in-depth investigation of these biological systems. For example, Mahadik et al. developed a novel microfluidic mixing platform for creating 3D hydrogel constructs that replicate the hematopoietic stem cell niche with overlapping gradients of cells and matrix components (Mahadik et al., 2014). This model enables detailed characterization and investigation of how microenvironmental signals influence hematopoietic stem cell behaviour. Similar studies could be carried out to extend the system to model the GP biology. For example, suitable techniques could be introduced to investigate cell-cell communication and cell-ECM interactions across different zones of the GP, as well as the cellular and molecular mechanisms underlying stem cell niche development by co-culturing chondrocytes with different cells located in the SOC.

In summary, by harnessing the potential of cutting-edge bioengineering strategies (e.g., biomimetic materials and innovative fabrication techniques), sophisticated *in vitro* models can be created, able to closely replicate the complex architecture and dynamic environment of the GP and its stem cell niche. This would in turn advance our understanding of the GP biology and pathology, and thus support the development of even higher fidelity models for the GP.

## 6 Conclusion

The development of a functional *in vitro* GP remains a significant challenge, despite considerable progress in understanding the cellular and molecular mechanisms governing its formation and maintenance. Current efforts have enabled generation of cartilage and bone organoids and successfully mimicked aspects of ECO, but the complexity of the GP architecture and its intricate molecular regulatory networks require further exploration. Future research must integrate cutting-edge bioengineering techniques with recent insights into chondrocyte differentiation, signaling pathways, and ECM interactions to overcome the current limitations. By continuing to bridge the gap between developmental biology and bioengineering, the creation of a functional *in vitro* GP is an ambitious but not unreachable goal that holds transformative potential for both research and clinical applications (Figure 1E).

Furthermore, the concept of engineering a functional GP will provide a paradigm for generating tissues with the intrinsic capacity of controlled growth. This could revolutionize pediatric regenerative medicine by enabling the development of bioengineered constructs capable of adapting and expanding in response to developmental cues. The concept could lead to novel therapeutic strategies for congenital and acquired defects such as skeletal dysplasia, growth plate injuries, and limb length discrepancies.
